# Association between chili pepper consumption and risk of gastrointestinal-tract cancers: A meta-analysis

**DOI:** 10.3389/fnut.2022.935865

**Published:** 2022-11-03

**Authors:** Changchang Chen, Man Zhang, Xutong Zheng, Hongjuan Lang

**Affiliations:** ^1^Department of Nursing, Fourth Military Medical University, Xi’an, China; ^2^School of Nursing, Yan’an University, Yan’an, China; ^3^School of Nursing, Fujian University of Traditional Chinese Medicine, Fuzhou, China

**Keywords:** chili pepper, gastrointestinal tract cancer, systematic review, meta-analysis, risk

## Abstract

**Background:**

Stimulating food is emerging as an important modifiable factor in the development of gastrointestinal (GI) tract cancers, but the association between chili pepper consumption and the risk of GI cancers is unclear. We aimed to evaluate the direction and magnitude of the association between chili pepper consumption and the risk of GI cancers.

**Methods:**

A literature search was performed in PubMed, Embase, and Web of Science databases from inception to 22 December 2021. Observational studies reporting the association between chili pepper consumption and the risk of gastric cancer (GC), esophageal cancer (EC), and/or colorectal cancer (CRC) in adults were eligible for inclusion. Data extraction and quality assessment were conducted independently by two reviewers for the included literature. Summary odds ratios (ORs) and 95% confidence intervals (CIs) were calculated using a random-effects model. Subgroup analyses were also performed based on the cancer type, study design, region of the study, study quality, and adjustments.

**Results:**

A total of 11,421 studies were screened, and 14 case-control studies were included involving 5009 GI cancers among 11,310 participants. The summary OR showed that high consumption of chili pepper was positively related to the risk of GI cancers (OR = 1.64; 95% CI: 1.00–2.70). A stronger positive relationship was observed between chili pepper consumption and EC risk (OR = 2.71; 95% CI: 1.54–4.75), but there was no statistically significant association between GC and CRC risk. In analyses stratified by geographical location, a positive association was found between chili pepper consumption and the risk of GI cancers in Asian studies (OR = 2.50; 95% CI: 1.23–5.08), African studies (OR = 1.62; 95% CI: 1.04–2.52), and North American studies (OR = 2.61; 95% CI: 1.34–5.08), but an inverse association was seen in South American studies (OR = 0.50; 95% CI: 0.29–0.87) and European studies (OR = 0.30; 95% CI: 0.15–0.61).

**Conclusion:**

This meta-analysis suggests that chili pepper is a risk factor for certain GI cancers (e.g., EC). Geographical regions influence the risk of GI cancers, especially in Asian, African, and North American populations, which require more attention during dietary guidance.

**Systematic review registration:**

[https://www.crd.york.ac.uk/PROSPERO/], identifier [CRD42022320670].

## Introduction

Globally, gastrointestinal (GI) tract cancers are a significant cause of morbidity and mortality, of which the most prevalent are colorectal cancer (CRC), gastric cancer (GC), and esophageal cancer (EC), ranking third, fifth, and eighth in incidence, respectively, but second, fourth, and sixth in mortality in both sexes combined according to GLOBOCAN estimates for 2020 (1). Despite the availability of multiple therapeutic options such as radiation, chemotherapy, curative resection, and immunotherapy, the early signs of GI cancers are generally undetectable and identified at an advanced stage, leaving patients with limited treatment options, and a poor prognosis (2). Therefore, early identification of risk factors for GI cancers is of great significance to public health.

Diet plays a major role in the development of these diseases. Chili pepper is one of the major vegetables and spices consumed around the world (3). Chili peppers are rich in the bioactive component capsaicin (CAP), which has been reported to have diverse biological properties such as anti-obesity, anti-oxidant, and anti-inflammatory effects *in vitro* and *vivo* experiments (4–6). Increasing evidence suggests that CAP facilitates the growth and migration of esophageal squamous cell carcinoma (ESCC) and human colon cancer cells (7, 8). Because of the CAP content, the association between chili pepper consumption and the risk of GI cancers is unclear, and it is important to clarify this question from a public health perspective. Previous individual studies, however, have reported the association between chili pepper exposure and the risk of GI cancers, with controversial results. This may be explained by heterogeneity among the studies, which is attributed to differences in the methods of exposure assessment, study area, sample sizes, and adjustments. For instance, Galvan-Portillo et al. (9) conducted a study of 726 subjects in Mexico, adjusted for energy, age, sex, and education, and showed a positive association between chili pepper and GC risk, whereas Munoz et al. (10) included 191 participants in Italy and observed an inverse association between chili pepper and GC risk after adjustment for sex, age, area of residence, and education. This difference can lead to confusion among dietitians and the general public, as well as challenges in translating into dietary advice.

Previous meta-analyses focusing on the association between chili pepper consumption and GC risk have yielded conflicting findings. For example, most studies showed a positive effect on GC risk (11–13), and a meta-analysis performed by Chen et al. (14) reported a null association. However, to date, no systematic review has been published that specifically explored the relationship between GI cancer risk and chili pepper consumption. Additionally, the validity of the above meta-analyses has been questioned due to the inclusion of studies that mixed chili pepper with other foods (12, 13), used kimchi or CAP instead of chili pepper as the interesting exposure (11–13), and extracted effect estimates incorrectly (12, 14), thus an extensive systematic review and meta-analysis is needed to obtain a more accurate estimate.

Hence, we performed a systematic review and meta-analysis to evaluate the association between chili pepper consumption and the risk of GI cancers by combining all available data from eligible studies. When possible, we used meta-analysis to quantify the effects and explore the possible sources of heterogeneity among the studies.

## Methods

The study was complemented following Preferred Reporting Items for Systematic Reviews and Meta-Analyses (PRISMA) (15). This review was registered at PROSPERO as CRD42022320670.

### Search strategy

We searched PubMed, Embase, and Web of Science for studies in humans on the association between chili pepper consumption and the risk of GI cancers from inception until 22 December 2021, using (“spicy” OR “chili” OR “chilli” OR “pepper” OR “capsaicin” OR “paprika”) AND (“malignancy” OR “cancer” OR “carcinoma” OR “tumor” or “neoplasm”) as search terms. Additionally, references to relevant articles and recent reviews were manually searched to identify other eligible articles.

### Selection criteria

Studies that satisfied the following criteria were included in this meta-analysis: (1) participants were adults; (2) studies were observational (cohort, case-control, or cross-sectional studies); (3) information was available on the relationship between chili pepper consumption as the exposure of interest and the risk of EC, GC, and/or CRC as the outcome of interest; and (4) studies reported available risk estimates in the form of relative risk (RR), odds ratio (OR), or hazard ratio (HR) with 95% confidence intervals (CIs). When overlapping populations were included in multiple articles, only the most recent or largest population was used to avoid duplications.

Non-English articles were excluded. Studies were excluded if they were reviews, letters, posters, meetings, or conference abstracts. We also excluded studies that included patients with precancerous lesions as an outcome of interest, mixed chili pepper with other foods (e.g., hot pepper-soybean stew) as the exposure of interest, had a sample size of fewer than 20 cases, and were conducted on children or adolescents. Additionally, studies with insufficient data were excluded. All searches were performed independently by two authors (CC and MZ), and inconsistencies were resolved through discussion.

### Data extraction

Two investigators (CC and MZ) independently reviewed and performed the data extraction from all the included studies. The extracted characteristics and data were composed of the first author’s last name, publication year, country, study design (hospital case-control, population case-control, or cohort study), number of study populations and cases, mean/median age of participants, male ratio, assessment method of exposure, GI cancer type, risk estimates and corresponding 95% CI, and covariates adjusted in multivariate analysis. For studies reporting several multivariate-adjusted risk estimates, the risk estimates that were maximally adjusted for underlying confounders were the top priority for use. Any discrepancies during the data extraction process were determined through discussion with a third investigator (XZ).

### Assessment of study quality

The quality of the included studies was evaluated using a modified version of the Newcastle–Ottawa Quality Assessment Scale (NOS) (16) with a nine-star scoring system. The following items were taken into consideration: selection of the study groups (up to four stars), comparability of the study groups (up to two stars), and confirmation of chili pepper exposure (up to three stars). We considered NOS scores above or equal to the median as high-quality studies (low risk of bias) and those with NOS scores below the median were regarded as low-quality (high risk of bias) (17). The results of study quality were not used as exclusion criteria.

### Statistical analysis

The OR and 95% CI were identified as effect sizes to evaluate the association between chili pepper consumption and the risk of GI cancers. We used the maximally adjusted OR reported in the original research when the OR was directly available. Heterogeneity among the included studies was evaluated by *I*-square (*I*^2^) statistic (18). When *I*^2^ was greater than 50%, there was significant heterogeneity between studies, so a random-effects model was selected. Otherwise, a fixed-effects model was performed (19). Publication bias was evaluated through a combination of qualitative and quantitative approaches, involving funnel plots and the Egger regression test (20).

A sensitivity analysis was conducted using the leave-one-out method to determine the influence of a single study. Subgroup analyses were also performed to explore whether pooled risk estimates were affected by cancer subgroups (EC, GC, or CRC), study design (population-based or hospital-based case-control study), region of the study (Asian, African, North American, South American, or European studies), study quality (high-quality or low-quality studies), adjustment for alcohol intake (Yes or No), and adjustment for smoking (Yes or No). Statistical analyses were done using Stata 16.0 software (StataCorp LLC, College Station, TX, USA). All *P-*values were two-sided, with *P* < 0.05 considered statistically significant.

## Result

### Literature search

**FIGURE 1:**
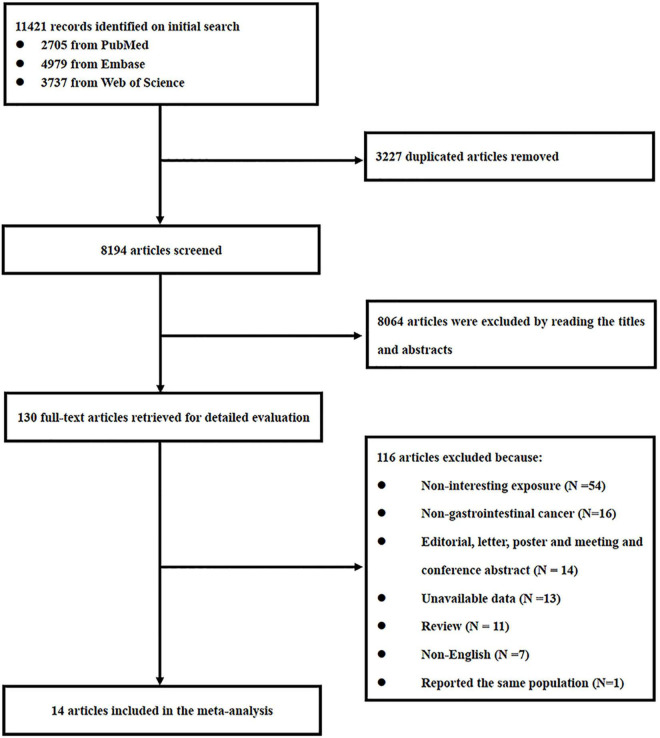
Flowchart of the study selection process.

Our search strategy retrieved 11,421 studies from 3 databases, and 3,227 duplicates were excluded. A further 8,194 studies were screened based on titles and abstracts, of which 8,064 articles were excluded because they did not meet the eligibility criteria. The remaining 130 studies were identified for full-text review, and 116 studies were excluded due to 54 being non-chili exposure, 16 being non-GI cancers, 14 studies being editorial, letter, poster, meeting, and conference abstract, the data of 13 studies being unavailable, 11 studies being review, 7 studies being published in a non-English language, and 1 study reporting the same population (21) with one of the included studies in this meta-analysis. In total, 14 studies (9, 10, 22–33) were included in our final analysis, and the flow diagram of the literature search is shown in Figure 1.

### Study characteristics

Table 1 summarizes the main characteristics of the included studies. The included studies, which were published between 1987 and 2021, included 14 case-control studies with 5009 GI cancers among 11,310 participants. The number of GI cases enrolled in these articles ranged from 87 to 833, and the number of participants ranged from 191 to 1666. Of the 14 case-control studies, eight studies were conducted in Asia (25–28, 30–33), two in Europe (10, 29), two in North America (9, 23), one in Africa (24), and one in South America (22). As to study design, most of these studies were population-based controls (9, 22, 23, 26, 30, 33), and the remaining six studies used a hospital-based case-control design (10, 24, 25, 27–29, 31, 32). Moreover, seven studies examined the association between chili pepper intake and the risk of GC (9, 10, 22, 23, 25, 27, 28), three on CRC (29, 32, 33), and four on EC (24, 26, 30, 31). In terms of the assessment methods of exposure, five studies (9, 22–24, 31) used the FFQ, while nine studies used frequency-reported questionnaires (10, 25–30, 32, 33). Almost all studies reported OR, except for two studies that reported RR (29, 30). All studies were adjusted or matched for age and sex, with only one study not adjusted for sex because all participants were male (30). Smoking (24, 25, 28, 31–33) and alcohol consumption (24, 25, 27, 28, 30–33) have been controlled in several studies.

**TABLE 1:** Characteristics of studies included in the meta-analysis.

References	Country	Study design	Sample sizes/cases	Age, male ratio	Assessment of exposure	Cancer type	Risk estimate (95% CI)	Adjustments
Liu et al. (32)	China	HCCS	1666/833	60 (53−67) vs. 60 (53−66), 58%	Q	CRC	Sweet pepper category: OR <0.75 kg/year: 1 0.75−2.60 kg/year: 0.54 (0.37−0.78) 2.60−5.20 kg/year: 0.52 (0.35−0.75) ≥5.20 kg/year: 0.48 (0.33−0.70)	BMI, colon cancer in first- degree relative, smoking status, alcohol drinking, eating breakfast, fried food, grilled food, hot and spicy food intake, total energy, total fruits, milk product, and red meat intake
Mmbaga et al. (24)	Tanzania	HCCS	942/471	59 (47−69) vs. 55 (45−65), 69%	FFQ	EC	Spicy chilies category: OR <daily: 1 Daily: 1.62 (1.04−2.52)	NA
Yang et al. (33)	China	PCCS	800/400	55.7 ± 11.08 vs. 55.74 ± 11.19, 58.2%	Q	CRC	Chili peppers category: OR ≤2 times/week: 1 3−7 times/week: 1.20 (0.75−2.00) > 7 times/week: 1.40 (0.84−2.20)	Intake of red meat, cured meat, pickles, tea, bean, fruit, vegetables, high-fat food, sweetmeats, daily sitting time, smoking regularly, drinking regularly, exercise regularly, and family history of CRC
Galván-Portillo et al, 2009 (9)	Mexico	PCCS	726/248	58 (mean), 54%	FFQ	GC	Chili category: OR No: 1 Regular: 1.19 (0.77−1.84) Much: 1.96 (1.26−3.05)	Energy, age, sex, and education
Goh et al. (27)	Malaysia	HCCS	261/87	61.4 ± 13.0 vs. 58.9 ± 10.8, 49%	Q	GC	Chili category: OR Low/none: 1 Heavy: 1.81 (0.74−4.43)	Race, *H. pylori* status, education, smoking, fresh fruits/vegetables, and salted fish/vegetables
Wang et al. (26)	China	PCCS	763/355	61.51 ± 7.94 vs. 60.75 ± 8.30, 62.3%	Q	EC	Chili category (men): OR Seldom: 1 Often: 3.38 (2.12−5.39) Chili category (women): OR Seldom: 1 Often: 1.61 (0.66−3.89)	Age, marital status, and education years
Phukan et al. (31)	India	HCCS	1506/502	55.0 ± 8.1 vs. 54.5 ± 7.8, NA	FFQ	EC	Chili category: OR Moderate user: 1 Non-user: 0.10 (0.05−5.80) Very chili: 3.60 (1.80−8.60)	Education, income, chewing betel nut and tobacco, smoking, and alcohol use
Muñoz et al. (22)	Venezuela	PCCS	777/292	> 35, NA	FFQ	GC	Chili category: OR Not often: 1 Often: 0.50 (0.30−0.90)	Age, sex, and socio-economic status
Mathew et al. (25)	India	HCCS	499/194	> 20, 76.0%	Q	GC	Chili category: OR Blank: 1 Medium: 1.80 (1.00−3.10) Very hot: 7.40 (4.00−13.50)	Age, sex, religion, education, smoking, and alcohol habits
López-Carrillo et al. (23)	Mexico	PCCS	972/220	57.2 vs. 59.2 (mean), 43.2%	FFQ	GC	Chili pepper (none of alcohol per day) category: OR No: 1 Yes: 4.50 (1.92−10.71) Chili pepper (<5 g of alcohol per day) category: OR No: 1 Yes: 2.90 (0.84−9.96)	Age, sex
Muñoz et al. (10)	Italy	HCCS	191/88	≤75, NA	Q	GC	Peppers category: OR 0 time/week: 1 1 time/week: 0.42 (0.21−0.86) ≥2 times/week: 0.31 (0.12−0.83)	Sex, age, area of residence, and education
Fernandez et al. (29)	Italy	HCCS	220/112	≤75, 57.7%	Q	CRC	Peppers category: RR Low: 1 Intermediate: 0.40 (0.20−0.70) High: 0.30 (0.10−0.70)	Sex, age, and area of residence
Gajalakshmi et al. (28)	India	HCCS	776/388	NA, 73.9%	Q	GC	Chilies category: OR Medium: 1 Hot: 2.80 (1.73−4.54)	Smoking, drinking alcohol, chewing habit, factors significant in the multivariate model of dietary item analysis, income group, educational level, and area of residence
Notani et al. (30)	India	PCCS	1211/819	NA, 100%	Q	EC	Red chili powder category: RR <75 g/cu/month: 1 75−99 g/cu/month: 1.94 (0.80−4.90) 100−149 g/cu/month: 1.99 (1.00−4.00) ≥150 g/cu/month: 2.85 (1.50−5.50)	Age, tobacco habits

GC, gastric cancer; EC, esophagus cancer; CRC, colorectal cancer; g/cu/month, grams per consumption unit per month; Q, questionnaire; FFQ, food frequency questionnaire; OR, odds ratio; RR, relative risk; CI, confidence interval; NA, not available; BMI, body mass index; HCCS, hospital case-control study; PCCS, population case-control study.

The detailed quality assessment of the included studies by the modified NOS for case-control studies is shown in Table 2. The median NOS score is 7. Eleven studies (9, 10, 21, 22, 24–26, 28–30, 33) with a NOS score of 7 or higher were evaluated as high methodological quality (low risk of bias), and three studies (27, 31, 32) with a score lower than 7 were assessed as low methodological quality (high risk of bias).

**TABLE 2:** Quality of studies according to the modified Newcastle-Ottawa Scale (NOS).

	Case-control studies													
	Liu et al. (32)	Mmbaga et al. (24)	Yang et al. (33)	Galván-Portillo et al. (9)	Goh et al. (27)	Wang et al. (26)	Phukan et al. (31)	Muñoz et al. (22)	Mathew et al. (25)	López-Carrillo et al. (23)	Muñoz et al. (10)	Fernandez et al. (29)	Gajalak-shmi et al. (28)	Notani et al. (30)
**Selection**														
1. Is the case definition adequate?	•	★	★	★	★	•	★	★	★	★	★	★	★	•
2. Representativeness of the cases	★	★	★	★	★	★	★	★	★	★	★	★	★	★
3. Selection of controls	•	•	★	★	•	★	•	★	•	★	•	★	•	★
4. Definition of controls	★	★	★	★	•	★	•	★	★	•	★	★	★	★
**Comparability**														
5. Study controls for the most important factor	★	★	★	★	★	★	★	★	★	★	★	★	★	★
6. Study controls for the second important factor	★	★	★	★	★	★	★	★	★	★	★	★	★	★
**Exposure**														
7. Was the measurement method of chili pepper described?	★	★	★	★	★	★	★	★	★	★	★	★	★	★
8. Were the methods of measurements same for cases and controls?	★	★	★	★	★	★	★	★	★	★	★	★	★	★
9. Non-response rate	•	★	★	•	•	★	•	•	•	•	•	•	•	•
**Summary score**	6/9	8/9	9/9	8/9	6/9	8/9	6/9	8/9	7/9	7/9	7/9	7/9	7/9	7/9
**(Risk of bias)**	(high)	(low)	(low)	(low)	(high)	(low)	(high)	(low)	(low)	(low)	(low)	(low)	(low)	(low)

★ was awarded when the respective information was available.

• was awarded if the respective information was unavailable.

### Chili pepper consumption and the risk of gastrointestinal cancers

**FIGURE 2:**
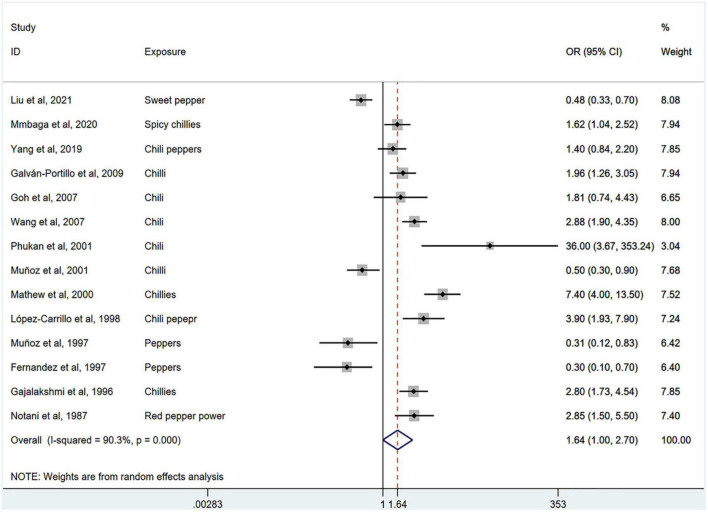
Pooled analysis showing associations between chili pepper consumption and the risk of GI cancers.

**FIGURE 3:**
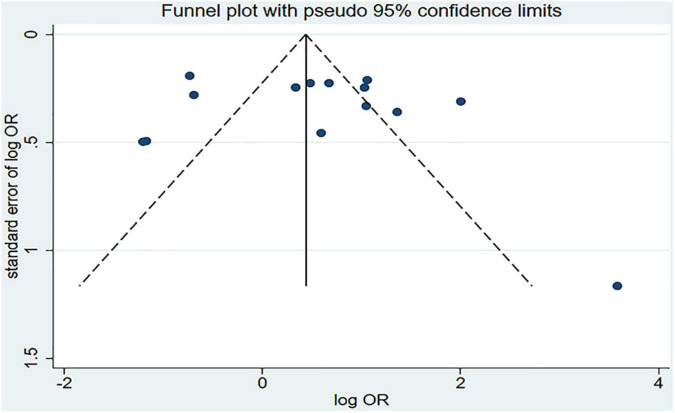
Funnel plot for evaluation publication bias. OR, odds ratio.

Figure 2 shows the results of the pooled analysis of the eligible studies. The association between chili pepper consumption and the risk of GI cancers was evaluated in 14 studies, consisting of 11,310 participants and 5009 cases. Pooled results showed that the highest category of chili pepper consumption was associated with an increased risk of GI cancers (OR = 1.64; 95% CI: 1.00–2.70), compared with the lowest category (or no intake). Significant heterogeneity existed across the studies (*I^2^* = 90.3%; *P* < 0.001). No evidence of publication bias was found based on Egger’s test (*P* = 0.651) and symmetrical funnel plot (Figure 3).

### Subgroup analysis

**FIGURE 4:**
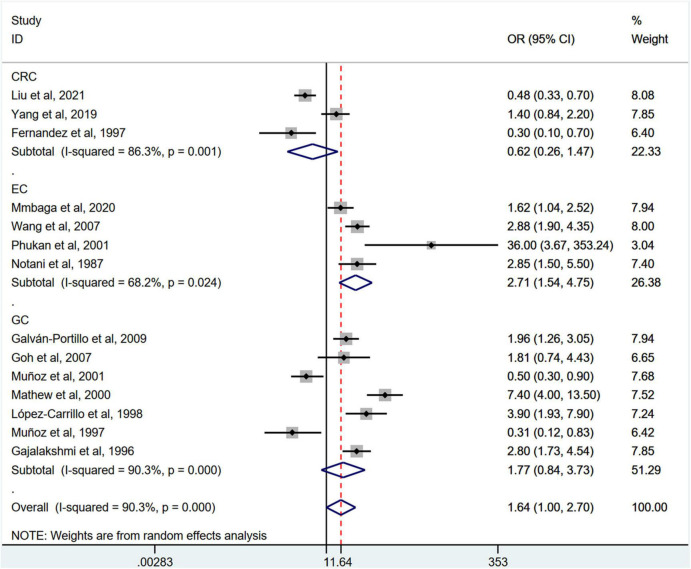
Subgroup analysis showing associations between chili pepper consumption and the risk of GI cancers based on the cancer type. GC, gastric cancer; EC, esophageal cancer; CRC, colorectal cancer; OR, odds ratio; CI, confidence interval.

**FIGURE 5:**
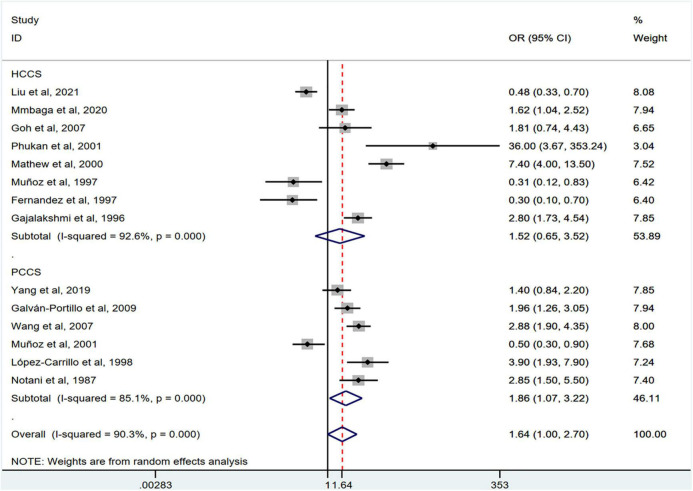
Subgroup analysis showing associations between chili pepper consumption and the risk of GI cancers based on the study design. HCCS, hospital case-control study; PCCS, population case-control study; OR, odds ratio; CI, confidence interval.

**FIGURE 6:**
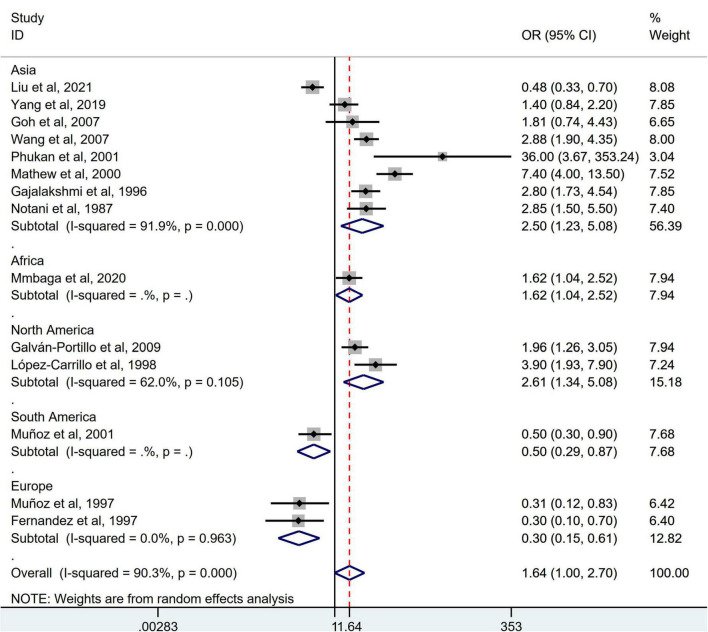
Subgroup analysis showing associations between chili pepper consumption and the risk of GI cancers based on the region of the study. OR, odds ratio; CI, confidence interval.

**FIGURE 7:**
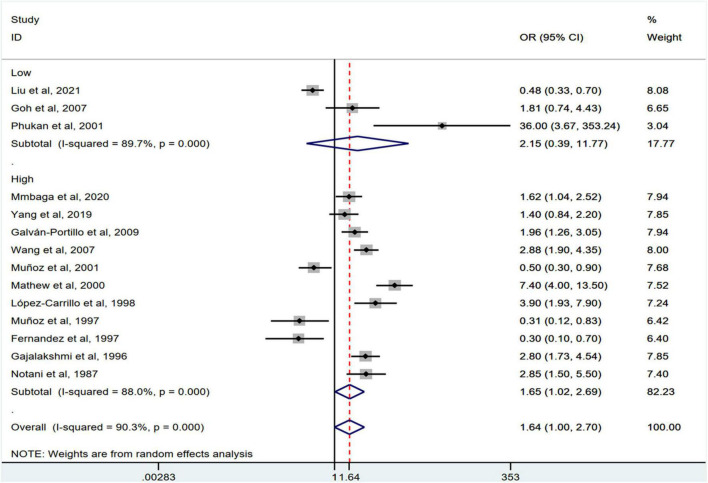
Subgroup analysis showing associations between chili pepper consumption and the risk of GI cancers based on the study quality. OR, odds ratio; CI, confidence interval.

**FIGURE 8:**
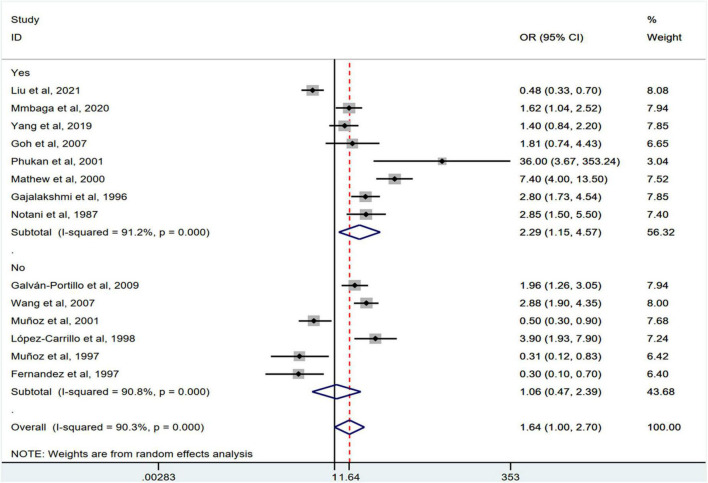
Subgroup analysis showing associations between chili pepper consumption and the risk of GI cancers based on the adjustment for alcohol intake.

**FIGURE 9:**
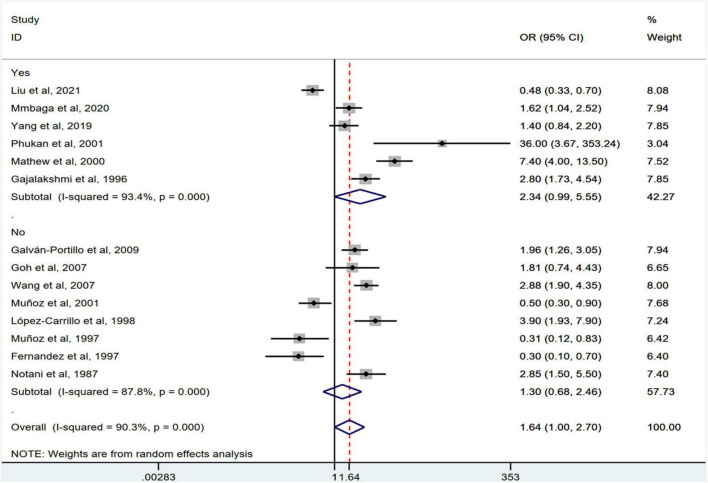
‘Subgroup analysis showing associations between chili pepper consumption and the risk of GI cancers based on the adjustment for smoking.

Stratification by cancer type showed that higher chili pepper consumption was associated with an elevated risk of EC (OR = 2.71; 95% CI: 1.54–4.75), but not with GC (OR = 1.77; 95% CI: 0.84–3.73) and CRC risk (OR = 0.62; 95% CI: 0.26–1.47) (Figure 4). When stratified by study design (Figure 5), population-based case-control studies showed a positive association between chili pepper consumption and the risk of GI cancers (OR = 1.86; 95% CI: 1.07–3.22), whereas hospital-based case-control studies showed a null association (OR = 1.52; 95% CI: 0.65–3.52). In the subgroup analysis of the region of the study (Figure 6), chili pepper consumption obviously increased the risk of GI cancers in Asian studies (OR = 2.50; 95% CI: 1.23–5.08), North American studies (OR = 2.61; 95% CI: 1.34–5.08), and African studies (OR = 1.62; 95% CI: 1.04–2.52). However, a significantly lower risk of GI cancers was observed in South American studies (OR = 0.50; 95% CI: 0.29–0.87) and European studies (OR = 0.30; 95% CI: 0.15–0.61). We further performed subgroup analysis by study quality (Figure 7) and adjustment factors (Figures 8, 9), finding a significant positive association between the highest chili pepper consumption compared with the lowest and the risk of GI cancers was seen in high-quality studies (OR = 1.65; 95% CI: 1.02–2.69), as well as in studies that adjusted for alcohol intake (OR = 2.29; 95% CI: 1.15–4.57). However, a null association was seen between chili pepper consumption and GI cancer risk in low-quality studies (OR = 2.15; 95% CI: 0.39–11.77), and studies not adjusted for alcohol intake (OR = 1.06; 95% CI: 0.47–2.39). A non-significant association was also seen in studies either adjusted for smoking or not.

## Discussion

This systematic review and meta-analysis was designed to evaluate the association between chili pepper consumption and the risk of GI cancers. The evaluation of 14 case-control studies involving 11,310 participants found a positive association between chili pepper consumption and the risk of GI cancers. In the subgroup analysis, this correlation between high chili pepper consumption and rising GI cancer risk was applied to EC, but not to GC and CRC. Especially in Asia, Africa, and North America, chili pepper intake showed a significant positive correlation with GI cancer risk.

In this study, the intake of chili pepper was positively associated with EC risk. The same finding was also observed for the consumption of chili pepper and GI cancer risk. Given that EC is a part of all GI cancers, the observed increased association with GI cancers appears to be related to EC. Several mechanisms could explain why higher chili pepper consumption was significantly associated with an increased risk of EC, but not with GC and CRC. The different effects could be due to differences in cancer sites. Chili peppers are rich in CAP, which has an intensely pungent flavor, further leading to a sensation of tingling and burning pain by stimulating transient receptor potential vanilloid 1 (TRPV1) (34–36). The stomach and intestine share a common endodermal origin and their epithelium is renewed more rapidly than that of the esophagus (37). Therefore, the stomach and colorectum are less affected than the esophagus. In addition, the differences are associated with different signaling pathways. Studies have shown that oral intake of CAP increases NF-κB expression (38). The methyldiazonium ion is the ultimate dimethylhydrazine (DMH) oncogenic metabolite, which is responsible for the methylation of DNA bases, leading to increased proliferation of colonic epithelial cells and triggering NF-κB activation (39, 40). NF-κB can exert numerous pro-tumorigenic functions, such as stimulating cell growth and inducing cell proliferation (41). Conversely, CAP also induces the expression of NF-κB inhibitors, of which the downregulation of Smad4 plays a role in the suppression of cell growth and invasion (42). This may explain why chili pepper is not associated with GC and CRC. For EC, several studies have demonstrated the carcinogenic effects of CAP on EC. For example, Huang et al. showed that thermo-TRPVs are functionally expressed in Eca109 and TE-1 ESCC cell lines. Hyperactivation of TRPV1 and TRPV4 facilitates the growth and/or migration of ESCC (8).

Several meta-analyses have investigated the relationship between the frequency of chili pepper consumption and GI cancer risk, with controversial results. When comparing the highest with lowest categories, most meta-analyses revealed a positive association between chili pepper intake and GC risk (11–13), while Chen et al. (14) showed a null association with the risk of GC. This discrepancy between different meta-analyses may be relevant to the inaccurate inclusion of the original literature. We investigated the eligibility of the studies included in previous meta-analyses, the results of which are summarized in Table 3. The aforementioned meta-analyses included studies analyzing CAP or kimchi instead of chili pepper as exposure (11–13), chili pepper mixed with other foods as exposure (12, 13), incorrect extraction of risk estimates (12, 14), and precancerous lesions rather than GI cancers as interesting outcomes (12), which may be considered as a limitation. Moreover, in a previous meta-analysis (13), the researchers inappropriately substituted continuous variables for categorical variables (highest vs. lowest) to calculate the effect estimates. To address these limitations, we performed a systematic review and meta-analysis by solely including studies that specifically reported chili pepper consumption as the exposure and GI cancers as the outcome.

**TABLE 3:** Eligibility survey of the original included literature on the relationship between chili pepper consumption and GI cancers.

Meta 1 (13)	Meta 2 (12)	Meta 3 (14)	Meta 4 (11)	Rationality of literature inclusion	Reasons
	Trujillo Rivera et al. (59)			Unreasonable	Examined the relationship between capsaicin consumption and gastric cancer
Al-qadasi et al. (60)		Al-qadasi et al. (60)		Reasonable	A case-control study investigating the association between chili pepper consumption and gastric cancer
	Wu et al. (61)			Unreasonable	Assessed the association between spicy food intake with precancerous lesion of gastric cancer
Xue et al. (62)				Reasonable	Chinese literature
Peng et al. (63)				Reasonable	Chinese literature
	López-Carrillo et al. (60)			Unreasonable	Examined the relationship between capsaicin consumption and gastric cancer
	Zhang et al. (65)			Unreasonable	Explored the relationship between kimchi and gastric cancer
Gómez Zuleta et al. (66)			Gómez Zuleta et al. (66)	Unclear	Non-english literature
Galván-Portillo et al. (9)			Galván-Portillo et al. (9)	Reasonable	A case-control study investigating the association between chili pepper consumption and gastric cancer
		Wang et al. (26)		Reasonable	A case-control study assessing the association between chili pepper consumption and esophageal squamous cell carcinoma cancer
Goh et al. (27)	Goh et al. (27)	Goh et al. (27)		Reasonable	A case-control study investigating the association between chili pepper consumption and gastric cancer
Bermúdez et al. (67)			Bermúdez et al. (67)	Unclear	Non-english literature
	Nan et al. (68)			Unreasonable	The study determining the risk relationship between kimchi and gastric cancer
López-Carrillo et al. (69)	López-Carrillo et al. (69)		López-Carrillo et al. (69)	Unreasonable	Examined the relationship between capsaicin consumption and gastric cancer
	Lee et al. (70)			Unreasonable	The study determining the risk relationship between kimchi and gastric cancer
Stefani et al. (71)				Unreasonable	Red pepper as a continuous variable rather than high versus low category
Muñoz et al. (22)			Muñoz et al. (22)	Reasonable	A case-control study investigating the association between chili pepper consumption and gastric cancer
		Phukan et al. (31)		Reasonable	A case-control study assessing the association between chili pepper consumption and esophageal cancer
Mathew et al. (25)	Mathew et al. (25)	Mathew et al. (25)		Reasonable	A case-control study investigating the association between chili pepper consumption and gastric cancer
Botterweck et al. (72)				Unreasonable	Red pepper as a continuous variable rather than high versus low category
López-Carrillo et al. (23)				Reasonable	A case-control study investigating the association between chili pepper consumption and gastric cancer
Gajalakshmi et al. (28)	Gajalakshmi et al. (28)	Gajalakshmi et al. (69)		Reasonable	A case-control study investigating the association between chili pepper consumption and gastric cancer
Lee et al. (73)	Lee et al. (73)			Unreasonable	The study measuring the association between hot pepper-soybean paste stew and gastric cancer
López-Carrillo et al. (21)	López-Carrillo et al. (21)	López-Carrillo et al. (21)	López-Carrillo et al. (21)	Reasonable	A case-control study investigating the association between chili pepper consumption and gastric cancer
		Notani et al. (30)		Reasonable	A case-control study investigating the association between chili pepper powder consumption and esophageal cancer
	Tajima et al. (74)	Tajima et al. (74)		Unreasonable	Incorrect risk estimates extracted

When stratified by the region of the studies, those studies conducted in Asia, North America, and Africa indicated that participants consuming the highest category of chili pepper had a greater risk of GI cancers, whereas three studies conducted in South America and Europe reported a significantly lower risk of GI cancers (10, 22, 29). One possible reason is that the number of included studies was relatively small, although an extensive search was done. Most original studies were conducted in Asian countries (25–28, 30–33), with only two in Europe (10, 29), two in North America (9, 23), one in Africa (24), and one in South America (22). The results should be cautiously interpreted. Moreover, most original studies reporting a lower risk of GI cancers were conducted in Europe. The evidence has shown that the estimated daily mean CAP intake in Europe was approximately 1.5 mg, which was less than the consumption level in Asia (e.g., Thailand) and North America (e.g., Mexico) (25–200 mg/person/day CAP) (43, 44). Therefore, the results of the highest than lowest or no chili pepper intake in studies conducted in Europe were more likely to obtain a protective effect, whereas the opposite effect was found in studies from Asia, North America, etc. Carcinogenicity or anticancer differences in chili pepper may depend on the dose. Further confirmation is needed to determine whether there is a U-shaped curve relationship, suggesting that a low dose of chili pepper intake might reduce GI cancer risk while a high dose intake might not.

In addition, subgroup analysis revealed that studies adjusted for alcohol consumption in the final model examining chili pepper intake and the risk of GI cancers had a positive association. Meanwhile, a seemingly stronger association between chili pepper consumption and the risk of GI cancers was observed in studies with adjustment for smoking than in those without such adjustment. The small number of original studies focusing on adjustment for smoking (n = 8) or alcohol consumption (n = 6) may be a possible reason. Another explanation is that numerous studies have found alcohol consumption or smoking to be related to a higher risk of GI cancers (45–48). However, there is currently no consensus on whether GI cancer risk is strongly associated with alcohol and smoking, because the evidence for heterogeneity by sex, age, cancer site, age at initiation, clinical stage of cancer, cancer grade, alcohol or smoking intensity, and duration is mixed (49–52). The mechanisms underlying the effects of alcohol consumption and smoking on GI cancers have not been comprehensively elucidated. These factors may affect the accuracy of the analysis. Regarding the study design, a significant association was found in the population-based case-control studies between chili pepper consumption and the risk of GI cancers but not in hospital-based case-control studies. The lack of representativeness may account for this difference.

Although a series of prespecified subgroup analyses were conducted, some heterogeneity generally persisted and could not be reduced. There were some other reasons for the heterogeneity among the included studies. First, the types of chili peppers consumed in different regions may have contributed to the heterogeneity among the results. However, few of the included studies reported specific types of chili peppers, except for two studies, in which the types of chili peppers were reported as sweet pepper (32) and red pepper powder (30), respectively. Second, stratified analysis of Helicobacter pylori (*H. pylori*) infection status was limited as data on *H. pylori* infection were only provided in one original study (27). *H. pylori* infection is a major risk factor for GI cancers. Experimental studies have suggested that combined *H. pylori* infection and CAP contribute to gastric inflammation and lead to GC with 50% incidence by regulating the expression of interleukin-6 (IL-6) and IFN-γ (53). On the other hand, chili pepper consumption may affect the *H. pylori* infection rate (54). Thus, *H. pylori* infection may act as a mediator and confound the association between chili pepper consumption and cancer risk.

Certain limitations of this study should be acknowledged. First, given the observational nature of the included studies, it is possible that the associations we found reflected residual confounding. Although a large number of potential confounders, such as cancer type, study design, and region of the study, were adjusted for in most studies, we cannot exclude that some other dietary biologically active components may partly or wholly affect the association. Second, recall bias associated with the assessment methods of chili pepper exposure should be considered because FFQ or frequency-reported questionnaires are subject to measurement errors, which can attenuate or overestimate the observed association (55). Additional limitations related to different cooking and processing methods for chili pepper. Several studies have examined various cooking methods (roasting, boiling, steaming, and stir-frying), cooking time, and temperature of chili pepper affect their phytonutrient content (56–58). However, the studies we included did not investigate the effect of chili pepper preparation methods, which prevented us from further exploring the sources of heterogeneity. Third, the dose–response analysis could not be conducted due to the insufficient number of available studies. Finally, only studies published in English were included, which may lead to the exclusion of related studies in other languages.

## Conclusion

Our results suggest that chili pepper consumption is associated with an increased risk of certain GI cancers. An increased EC risk was observed when high levels of chili pepper were ingested. However, no significant association was found between chili pepper consumption and the risk of GC and CRC. More prospective cohort studies are necessary to clarify the dose-response effect of chili pepper on the risk of GI cancers.

## Data availability statement

The original contributions presented in this study are included in the article/Supplementary material, further inquiries can be directed to the corresponding author.

## Author contributions

CC and MZ designed the study, performed the literature search, extracted data, conducted the statistical analysis, and drafted the manuscript. CC, XZ, and HL performed data screening, interpreted the data, and performed the writing review of the manuscript. All authors contributed to the articles and approved the final manuscript.
