# Supplementary material for: Association between chili pepper consumption and risk of gastrointestinal-tract cancers: A meta-analysis
Source: Front Nutr. 2022 Nov 3;9:935865. doi: 10.3389/fnut.2022.935865 (PMC9669750; doi:10.3389/fnut.2022.935865)

**Supplementary Figure 1.**  Sensitivity analysis of chili pepper consumption and gastrointestinal tract cancers.


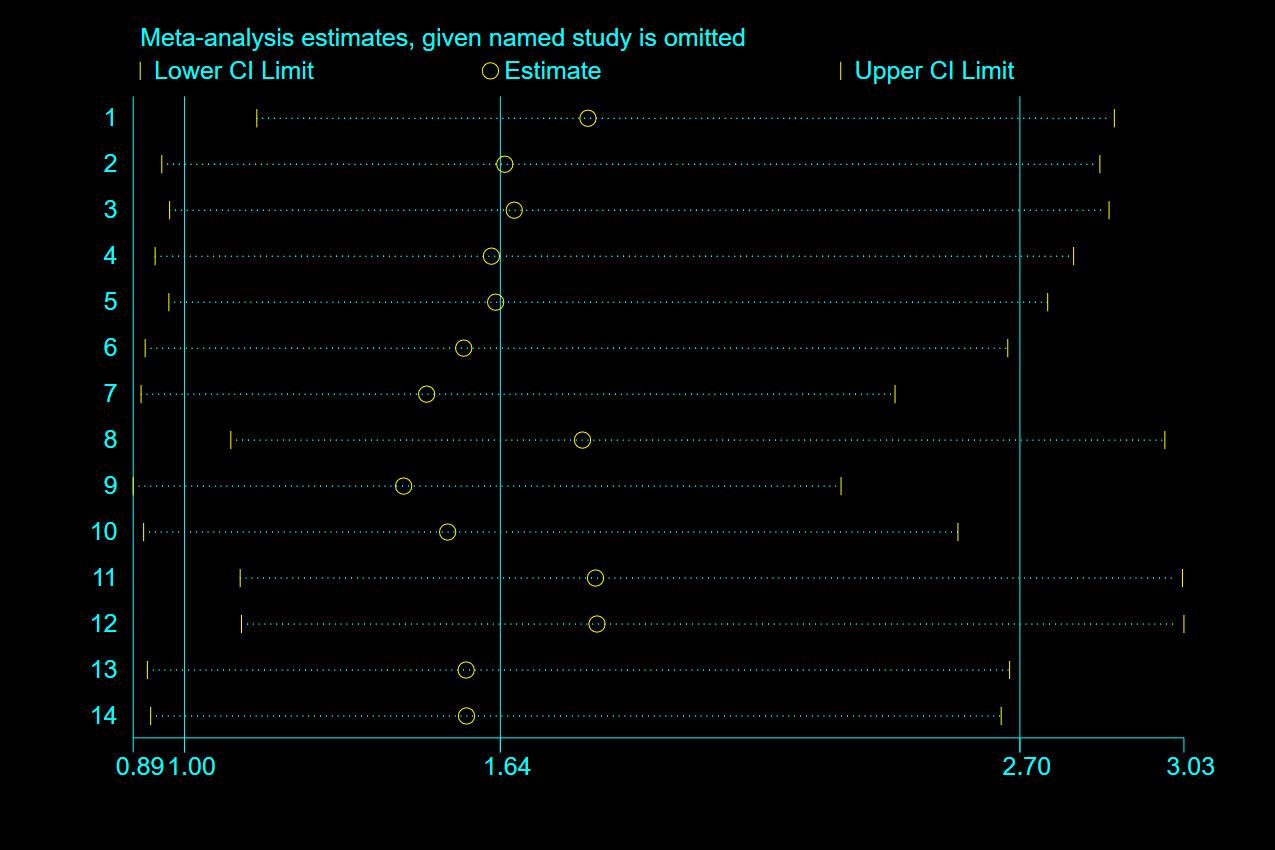

Supplement: Supplementary file 1 [file Data_Sheet_1.docx]
